# Quality of Life with Voice Prosthesis after Total Laryngectomy

**DOI:** 10.22038/ijorl.2021.53724.2832

**Published:** 2021-09

**Authors:** Nicola Massaro, Barbara Verro, Giuseppe Greco, Enzo Chianetta, Aurelio D'Ecclesia, Carmelo Saraniti

**Affiliations:** 1 *Department of Biomedicine, Neurosciences and Advanced Diagnostic, University of Palermo, Palermo (PA) Italy.*; 2 *ENT and Maxillo-Facial Clinic, IRCSS – Casa Sollievo Della Sofferenza – San Giovanni Rotondo, Foggia (FG), Italy.*

**Keywords:** Quality of life, Prostheses, Voice quality, Laryngectomy

## Abstract

**Introduction:**

The loss of voice after total laryngectomy is one of the main impairments in personal and social life. In order to prevent potential psycho-social consequences in the patient and his family, the restoration of phonatory function is the main objective of post-laryngectomy rehabilitation. The aim of this study was to assess quality of life in patients who received prosthetic voice after total laryngectomy.

**Materials and Methods:**

Over a one-year period, 51 patients with voice prostheses after total laryngectomy were recruited. 32 patients (62.74%) were administered radiation therapy and 9 patients (17.64%) underwent to surgical reconstruction with flaps. Each patient was administered the VHI-10 and V-RQOL self-assessment questionnaires.

**Results:**

The study showed that vocal restoration with voice prosthesis allows patients to recover a significant degree of quality of life after total laryngectomy. The average score on the V-RQOL questionnaire was 75.9 and on the VHI-10 questionnaire was 13.5. It has not been shown a statistically significant correlation between quality of life after tracheoesophageal prosthesis and radiation therapy, chemotherapy or reconstruction flaps. Younger patients showed, on average, a higher score at V-RQOL. These results allow to state that, after prosthetic rehabilitation, at least 75% of patients experienced an increase in quality of life. Moreover, the prosthetic technique (primary vs secondary) does not affect the long-term outcome and radiotherapy, chemotherapy or reconstruction flaps are not absolute contraindications to rehabilitation with voice prosthesis.

**Conclusion:**

After total laryngectomy, rehabilitation with tracheoesophageal prosthesis is a satisfactory choice to restore the patient’s ability to communicate verbally.

## Introduction

The loss of voice after total laryngectomy is one of the main impairments in personal and social life. This event drastically compromises the patient's oral communication and his integration and interaction in the social life (1,2). Indeed, the treatment of laryngeal cancer must consider not only the oncological outcome in terms of Overall Survival and Disease-Free Survival, but also the quality of life, especially communication and social integration (3). In order to prevent potential psycho-social consequences in the patient and his family, the restoration of the phonatory function is the main objective of post-laryngectomy rehabilitation. In particular, to date, the tracheoesophageal prosthesis represents the gold standard for voice restoration after total laryngectomy (4-8), improving quality of life after total laryngectomy (9). 

The Tracheo-Esophageal Puncture (TEP) can be performed at the end of laryngectomy (primary technique) or in a second step (secondary technique). Furthermore, for some patients who received radiation therapy before or after laryngectomy, the current literature reports an increased occurrence of complications such as wound infection and/or dehiscence that could impair tracheoesophageal prosthesis management (3,10). 

Complications occur more often in secondary prosthesis placement after radiotherapy, for this reason the tracheoesophageal prosthesis should be preferably placed at the end of laryngectomy or before radiation therapy (3). 

Thus, the aim of this study was to examine and assess the quality of life in patients who received prosthetic voice restoration after total laryngectomy. Another goal of this study was to quantify the degree of phonatory handicap subjectively perceived by patients using VHI-10 (11) and V-RQOL (12) questionnaires. 

The former is a useful tool that allows to quantify handicap related to own voice disorder and functional outcome after voice prosthesis placement. 

The latter questionnaire is a clinical tool that assesses the impact of voice disorder in patient’s quality of life. Finally, the team assessed whether the quality of life in patients with voice prosthesis was affected by chemo-radiation therapy or by surgical reconstruction with flaps during total laryngectomy.

## Materials and Methods

An observational study was accomplished using the VHI-10^11^ and V-RQOL^12^ questionnaires in patients who underwent total laryngectomy for laryngeal cancer, with subsequent phonatory rehabilitation using prosthesis. The study patients were recruited at the Otorhinolaryngology Department of the Paolo Giaccone University Hospital, in Palermo, between July 2018 and June 2019.

The eligibility criteria were: 1) patients over 18 years of age, 2) both males and females, 3) patients undergoing total laryngectomy, associated or not with radiotherapy, chemotherapy or flap reconstruction, due to laryngeal carcinoma, 4) patients with voice prostheses, with primary or secondary technique. The exclusion criteria were: 1) patients unable to understand and answer questionnaires, 2) patients with cognitive function impairments. This study was approved by the Ethical Committee of the University Hospital in Palermo.

In addition to the administration of the VHI-10^11^ and V-RQOL (12) self-assessment questionnaires, each patient was asked to provide the following data: 1) any peri radiation therapy or chemotherapy, 2) the type of technique used for the tracheoesophageal puncture (primary vs secondary), 3) use of reconstruction flaps during total laryngectomy. 

**Statistical analyses**

Data were statistically analyzed. In particular, Shapiro-Wilk normality test was used to verify whether two datasets (V-RQOL scores) had a normal distribution, according to a Gaussian curve. Then, a parametric test such as unpaired Student's t-test was used to compare these two values. Moreover, correlation between more factors was verified by calculating Spearman's rank correlation coefficient. A p-value <0.05 was considered significant.

## Results

51 laryngectomized patients who received a voice prosthesis were enrolled in this study. Table 1 shows the general characteristics of our cohort of patients, with the relative scores recorded in each of the two questionnaires. The patients are listed according to the time sequence of participation in this study.

**Table 1:** Overall characteristics of the patient cohort

**Patient**	**Age Of Laryngectomy **	**Laryngectomy**	**Tep**	**Radiation Therapy**	**Chemotherapy**	**Flaps**	**Vhi-10**	**V-Rqol**
1	64	2018	P				12	87.5
2	37	2005	S	X		X	16	75
3	53	2008	S				15	72.5
4	55	2012	S				5	97.5
5	47	2012	S			X	3	95
6	56	2009	S	X		X	8	92.5
7	64	2011	S	X	X		11	62.5
8	65	2012	S				12	65
9	39	2000	S				6	95
10	64	2009	S				28	50
11	69	2014	S	X	X		16	67.5
12	75	2014	S				20	60
13	53	2014	S	X	X		9	77.5
14	70	2016	S				21	77.5
15	55	2016	S	X			29	60
16	69	2018	P	X			37	32.5
17	79	2011	S	X			17	55
18	69	2010	S	X	X		14	67.5
19	54	2012	P				3	87.5
20	66	2014	S				26	35
21	50	2006	S	X		X	18	72.5
22	59	2012	P	X	X		13	70
23	56	2000	P	X			9	82.5
24	72	2015	P	X		X	14	70
25	72	2016	P	X			6	95
26	67	2019	P				16	72.5
27	79	2018	P	X	X		8	90
28	69	2018	P	X			8	90
29	69	2016	S	X			6	100
30	59	2017	S	X	X	X	10	95
31	67	2015	S	X			16	60
32	71	2015	S	X			12	75
33	46	2010	P				9	90
34	70	2009	S	X	X		10	90
35	69	2013	S	X			7	85
36	49	2006	S	X			12	75
37	71	2011	S	X			17	47.5
38	75	2011	S				14	60
39	69	2019	P	X			7	90
40	80	2018	P	X			16	77.5
41	43	2013	S				4	97.5
42	54	2017	P	X	X		22	65
43	73	2016	S				29	62.5
44	50	2008	S	X	X		15	80
45	77	2016	S				13	80
46	53	2015	S	X	X	X	23	60
47	63	2017	P	X	X		12	95
48	54	2013	P	X			3	87.5
49	79	2012	S	X	X	X	19	60
50	57	2010	P	X	X	X	8	87.5
51	46	2012	P				3	95

*Age Of Laryngectomy = age at time of total laryngectomy; TEP = tracheoesophageal puncture; P = primary; S = secondary; X = YES*

**V-RQOL questionnaire scores**

**Fig 1:**
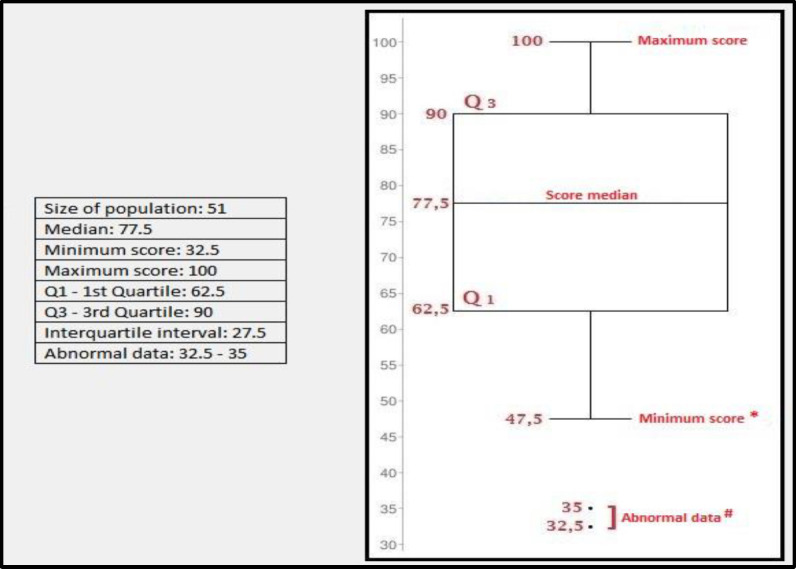
Box-Plot of the distribution of V-RQOL questionnaire scores. Q_1_: 1st Quartile; Q_3_: 3rd Quartile * Minimum score that does not differ from the median by more than one and a half times the interquartile range. # They differ from the median more than one and a half times the interquartile range

The results obtained from the administration of the V-RQOL questionnaire are shown in Table 2. Figure 1 shows the graphic distribution of the scores obtained by box plot. Taking into account the value of the 1st and 3rd Quartile, the diagram shows that 50% of the patients examined reported a score between 62.5 and 90. Since the third quartile (or 75th percentile - Q3) is equal to 90, it means that a further 25% of the sample scored at V-RQOL between 90 and 100. The value of the interquartile range was 27.5, and only 2 of the 51 scores (32.5 and 35) differed from the median by more than one and a half times the interquartile range, thus representing isolated cases compared with the rest of the distribution.

**Table 2:** V-RQOL questionnaire

**Average Scores**	**Median Score **	**Standard Deviation**	**Range**
75.9	77.5	16.3	32.5 -100

**VHI-10 questionnaire scores**

**Fig 2:**
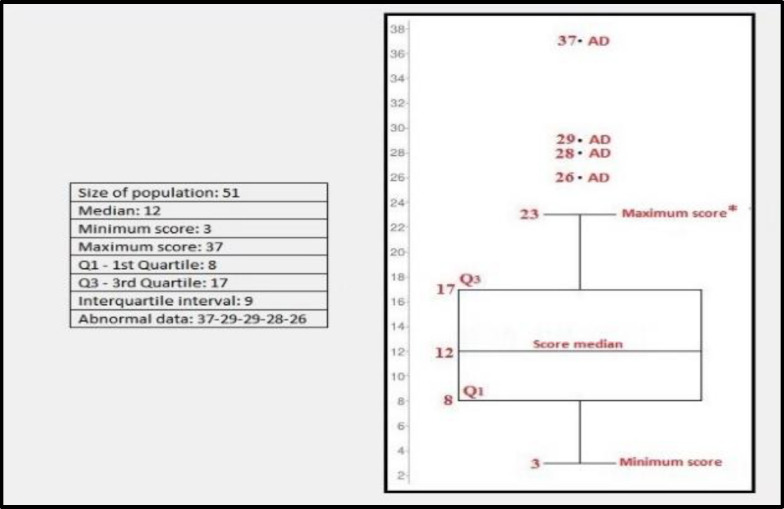
Box-Plot of the distribution of VHI-10 questionnaire scores Q_1_: 1st Quartile; Q3: 3rd Quartile

The results obtained from the administration of the VHI-10 questionnaire are shown in Table 3. Taking into account the median in the graph (Figure 2), it can be observed that 50% of the patients examined reported a score at VHI-10 between 3 and 12. Moreover, since the third quartile (or 75th percentile - Q3) is equal to 17, it can also be said that an additional 25% of the patients had a score between 12 and 17. The value of the interquartile range was 9, and 5 of the 51 scores (28 and 37) differed from the median by more than one and a half times the interquartile range, thus representing isolated cases compared with the rest of the distribution.

* Maximum score that does not differ from the median by more than one and a half times the interquartile range

AD = Abnormal Data: differ from the median by more than one and a half times the interquartile range

**Table 3:** VHI-10 questionnaire

**Range 0-10** **No/minimal handicap**	**Range 11-20** **Moderate handicap**		**Range 21-40** **Severe handicap**
20 PATIENTS (39.2%)	23 PATIENTS (45.1%)		8 PATIENTS (15.7%)
**Average Scores**	**Score Median**	**Standard Deviation**	**Range**
13.5	12	7.5	3-37.5

**Correlation between VHI-10 scores and matching V-RQOL scores**

**Fig 3:**
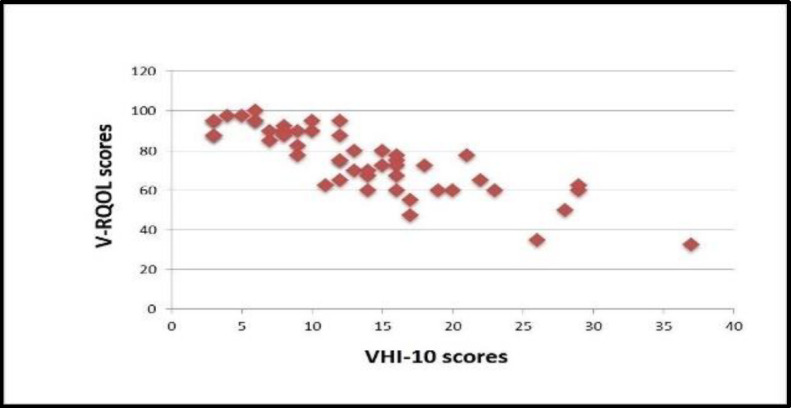
VHI-10 score scatter plot as a fuction of the respective V-RQOL scores

The dispersion graph (Figure 3) shows an inverse linear monotonic relationship between the VHI-10 and V-RQOL scores, i.e., patients with low VHI-10 tend to have a high V-RQOL score. This association was verified by calculating Spearman's rank correlation coefficient, and the data obtained for the cohort of our study are as follows: r_s_ = ρ(rho) = -0.8471 (P-value = 0.000). These calculations showed that there was an inversely proportional correlation, highly significant for statistical purposes, between the VHI-10 and V-RQOL scores.

**Age of patients at the time of prosthesis placement**

**Fig 4:**
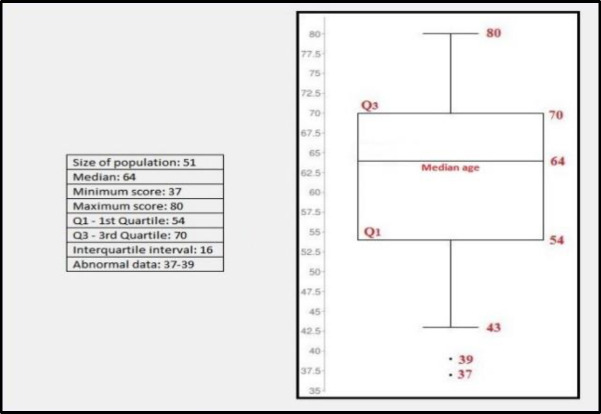
Box-Plot of the age distribution of patients at the time of voice prosthesis placement

The scatter plot in Figure 4 shows that 50% of the patients were between 54 and 70 years of age at the time of prosthesis placement. The median age of the sample was 64 years. In order to investigate a possible correlation between V-RQOL and age at prosthesis placement, the study cohort was divided into two groups, as indicated below: 

Group 1: aged 64 years old or younger; 

Group 2: aged over 64 years old.

The average value of the scores obtained in the V-RQOL questionnaire was then calculated in each of the two groups (Table 4).

**Table 4:** Average value of the scores obtained in the V-RQOL questionnaire

**Average Of V-Rqol Group 1**	**Average Of V-Rqol Group 2**
80.96	70.60

Group 1: patients aged less than or equal to 64 years; Group 2: patients older than 64 years

Later on, it was evaluated whether the difference between the averages of the two groups was statistically significant. First, the normal distribution of the two datasets (V-RQOL scores) was evaluated using the Shapiro-Wilk normality test. So, the unpaired Student's t-test was used and the result showed a statistically significant difference between the averages of two groups (two-tailed p-value 0.02) with a 95% significance level.

**Radiation therapy and quality of life after voice restoration with voice prosthesis**

**Fig 5:**
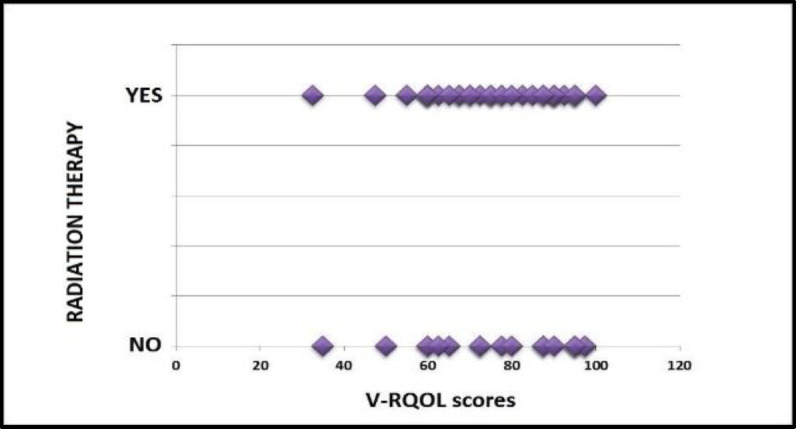
Scatter plot: scores obtained at V-RQOL as a function of the presence/absence of radiation therapy

We also determined whether performing radiation therapy in the peri-operative period could affect the subsequent V-RQOL after phonatory rehabilitation. Therefore, the population was first divided into two groups: patients undergoing peri-operative radiotherapy (group A) and patients not undergoing peri-operative radiotherapy (group B). The two datasets (V-RQOL scores) had a Gaussian curve. The unpaired Student's t-test showed the absence of a statistically significant difference between group A and group B in relation to V-RQOL scores (two-tailed p-value 0.80). This lack of correlation can also be seen in the scatter plot in Figure 5.

**Adjuvant chemotherapy and quality of life after voice restoration with voice prosthesis**

**Fig 6:**
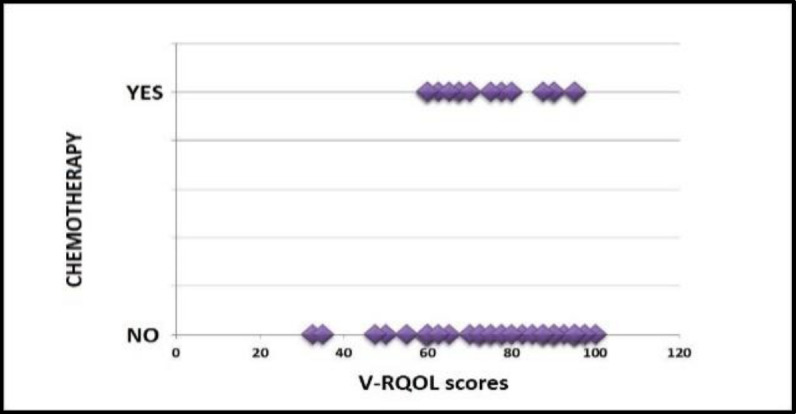
Scatter plot: scores obtained at V-RQOL as a function of the presence/absence of adjuvant chemotherapy

Subsequently, it was investigated whether adjuvant chemotherapy could affect the V-RQOL after phonatory rehabilitation. The cohort was divided into two group: patients undergoing peri-operative radiotherapy (group C) and patients not undergoing peri-operative radiotherapy (group D). After evaluating the normal distribution of the two datasets (V-RQOL scores) using the Shapiro-Wilk normality test, the unpaired Student's t-test was performed: a statistically significant difference between group C and group D in relation to V-RQOL scores was no found (two-tailed p-value 0.92). This lack of correlation can also be seen in the scatter plot in Figure 6.

**Reconstructive surgical flaps and quality of life after voice restoration with voice prosthesis**

**Fig 7:**
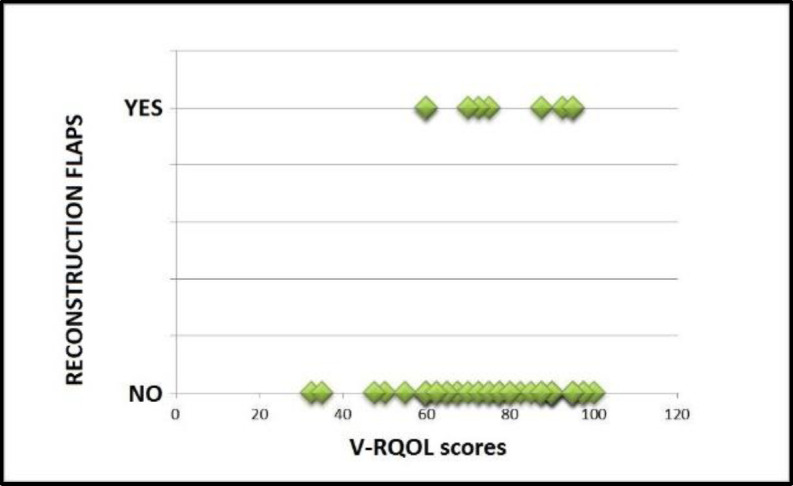
Scatter plot: scores obtained at V-RQOL as a function of the presence/absence of reconstruction flaps

Possible correlations were investigated between the use of reconstructive surgical flaps and the V-RQOL questionnaire score. Patients were grouped into two categories: use of reconstructive surgical flaps (group E) and non-use of reconstructive surgical flaps (group F). The two datasets (V-RQOL scores) had a Gaussian curve. The unpaired Student's t-test showed the absence of a statistically significant difference between group E and group F in relation to V-RQOL scores (two-tailed p-value 0.58). The lack of correlation is represented graphically in the scatter plot in Figure 7.

**Primary vs** **secondary** **technique** **and V-RQOL scores**

Among the totality of patients, 18 received a prosthesis with primary technique (group G) and 33 with secondary technique (group H). The two datasets (V-RQOL scores) had a normal distribution and a statistically significant difference between group G and group H in relation to V-RQOL scores was no found (two-tailed p-value 0.07).

## Discussion

**Summary of the main results**

Rehabilitation of laryngectomized patients with tracheoesophageal prosthesis is a satisfactory choice to give the patient a good quality of life in terms of verbal communication (4-6,8,9,13,14). 

The simplicity and reproducibility of the surgical techniques, the reduction of long-term complications and the continual improvement of the biomaterials used to make prostheses are leading to a broader application of this rehabilitation method (15-16). Patients should be carefully and rigorously studied before being admitted to prosthetic rehabilitation - to assess their mental profile and to determine their cognitive abilities (7,17-18). 

Our study shows that voice restoration with voice prosthesis allows rehabilitated patients after total laryngectomy to recover an acceptable quality of life. Overall, the VHI and V-RQOL scores in our cohort were on average lower than the general population but significantly higher than those of patients who did not receive prosthetic placement after total laryngectomy, as evidenced in previous studies (4,5,19,20). 

Moreover, our experience demonstrates that quality of life increases more in younger people: this may be due to a lower incidence of comorbidity in this age group. 

**V-RQOL questionnaire scores**

In this study, the average V-RQOL questionnaire score was 75.9, with a standard deviation of 16.1. This is significantly higher when compared with average V-RQOL scores in laryngectomized patients who did not receive phonatory rehabilitation (21-23). 

Moreover, about the statistical analysis of the distribution of the V-RQOL questionnaire scores, we observed that 50% of the patients examined reported a score between 62.5 and 90: this shows, statistically, that at least one out of every two patients has a V-RQOL ranging from “satisfactory" to "very good." Since the third quartile (or 75th percentile - Q3) is equal to 90, it means that a further 25% of the sample has a V-RQOL score between 90 and 100. This finding indicates a voice-related quality of life ranging from "very good" to "excellent,” and overlaps completely V-RQOL scores of healthy population, with average V-RQOL scores between 94.8 and 98 (12,24). 

Only 2 out of the 51 scores of our cohort (scores: 32.5 and 35) were classified as “abnormal data" because they differ from the median by more than one and a half times the interquartile range: however, these data are statistically isolated cases. Overall ¾ of the patients of our cohort have at least an acceptable quality of life, with scores ranging from “satisfactory" to "excellent" (scores similar to those of the healthy population). 

**VHI-10 questionnaire scores**

Data from the VHI-10 questionnaire showed that: 20 patients obtained a score of less than 10, which reveals absence of phonatory handicap or minimum handicap; 23 patients scored between 11 and 20, showing a moderate phonatory handicap; a severe phonatory handicap was found in 8 of the 51 patients with a score between 20 and 40. Taking into account the statistical analysis of the distribution of the VHI-10 questionnaire scores, the median was 12. This finding means that 50% of the enrolled patients reported a score at VHI-10 between 3 and 12, and so at least one out of every two patients did not have a phonatory handicap or had only a minimum phonatory handicap. Indeed, according to the average normal values at VHI-10 (Arffa et al (25), 2012), a score at VHI-10 greater than or equal to 11 can certainly be considered abnormal. Moreover, since the third quartile (or 75th percentile - Q3) is equal to 17, it can be stated that an additional 25% of our cohort had a moderate phonatory handicap. Only 8 patients out of 51 (15.7%) had a VHI-10 score more than 21 (severe phonatory handicap). The value of the interquartile range was 9, and 5 of the 51 scores were classified as “abnormal data" because they differ from the median by more than one and a half times the interquartile range (26,28,29,37), and therefore represent isolated cases. Moreover, this study demonstrated an inverse linear monotonic relationship between the VHI-10 scores and the matching V-RQOL scores; i.e., patients with a low VHI-10 have a high V-RQOL score. This finding is consistent with the relevant scientific literature (25-28).

**Age of patients at the time of prosthesis placement**

The comparison between the scores for the two age groups (patients aged less than or equal to 64 years vs patients older than 64 years) showed a statistically significant difference (two-tailed p-value 0.02). 

This finding indicates that, statistically, patients younger than 64 had a higher V-RQOL questionnaire score (average: 80.96) than the older population. Nonetheless, the average V-RQOL score (70.6) of the older patient group was significantly higher than the laryngectomized patients without phonatory rehabilitation, whose scores was in the 43.45-54.20 range (Rossi et al (21), 2014, Weinstein et al^22^, 2001). 

**Radiation therapy / Adjuvant chemotherapy / Reconstructive surgical flaps and quality of life after voice restoration with voice prosthesis**

The study did not show a statistically significant correlation between quality of life after TEP with voice prosthesis and radiation therapy, chemotherapy or use of reconstruction flaps, as reported in literature (2,3,29,30). 

**Primary vs** **secondary** **technique** **and V-RQOL scores**

Among the overall number of patients, 18 received a prosthesis with primary technique and 33 with secondary technique. The analysis carried out showed that the total score of each patient was not affected by the timing of prosthesis placement (primary vs secondary). This finding is compatible with current literature (8,13,31). 

## Conclusion

This study demonstrated the important role of voice prosthesis rehabilitation after total laryngectomy in terms of functional outcome (VHI score) and quality of life (V-RQOL score). The study also demonstrated that radiotherapy, chemotherapy and / or use of surgical reconstructive flaps do not statistically significantly affect voice-related quality of life. Therefore, TEP represents a satisfactory and effective choice for phonatory rehabilitation after total laryngectomy, since at least 75% of patients have a significant improvement in quality of life. 
